# Differences in musculoskeletal health due to gender in a rural multiethnic cohort: a Project FRONTIER study

**DOI:** 10.1186/s12891-016-1042-7

**Published:** 2016-04-26

**Authors:** J. M. Brismée, S. Yang, M. E. Lambert, M. C. Chyu, P. Tsai, Y. Zhang, J. Han, C. Hudson, Eunhee Chung, C. L. Shen

**Affiliations:** Center for Rehabilitation Research, Texas Tech University Health Sciences Center, Lubbock, TX 79430 USA; Department of Pathology, Texas Tech University Health Sciences Center, Lubbock, TX 79430 USA; F. Marie Hall Institute for Rural and Community Health Research, Texas Tech University Health Sciences Center, Lubbock, TX 79430 USA; Graduate Healthcare Engineering, Whitacre College of Engineering, Texas Tech University, Lubbock, TX 79409 USA; Department of Public Health, Texas Tech University Health Sciences Center, Lubbock, TX 79430 USA; Department of Family and Community Medicine, Texas Tech University Health Sciences Center, Lubbock, TX 79430 USA; Department of Nutrition and Food Hygiene, Xinjiang Medical University, Xinjiang, China; Department of Kinesiology and Sport Management, Texas Tech University, Lubbock, USA

**Keywords:** Gender, Hand grip strength, Gait, Musculoskeletal discomfort, Rural health, FRONTIER project

## Abstract

**Background:**

Very few studies have investigated differences in musculoskeletal health due to gender in a large rural population. The aim of this study is to investigate factors affecting musculoskeletal health in terms of hand grip strength, musculoskeletal discomfort, and gait disturbance in a rural-dwelling, multi-ethnic cohort.

**Methods:**

Data for 1117 participants (40 years and older, 70 % female) of an ongoing rural healthcare study, Project FRONTIER, were analyzed. Subjects with a history of neurological disease, stroke and movement disorder were excluded. Dominant hand grip strength was assessed by dynamometry. Gait disturbance including stiff, spastic, narrow-based, wide-based, unstable or shuffling gait was rated. Musculoskeletal discomfort was assessed by self-reported survey. Data were analyzed by linear, logistic regression and negative binomial regressions as appropriate. Demographic and socioeconomic factors were adjusted in the multiple variable analyses.

**Results:**

In both genders, advanced age was a risk factor for weaker hand grip strength; arthritis was positively associated with musculoskeletal discomfort, and fair or poor health was significantly associated with increased risk of gait disturbance. Greater waist circumference was associated with greater musculoskeletal discomfort in males only. In females, advanced age is the risk factor for musculoskeletal discomfort as well as gait disturbance. Females with fair or poor health had weaker hand grip strength. Higher C-reactive protein and HbA1c levels were also positively associated with gait disturbance in females, but not in males.

**Conclusion:**

This cross-sectional study demonstrates how gender affects hand grip strength, musculoskeletal discomfort, and gait in a rural-dwelling multi-ethnic cohort. Our results suggest that musculoskeletal health may need to be assessed differently between males and females.

## Background

Musculoskeletal health involves muscle strength, bone strength, and joint health. A decrease in bone mass and strength, and an increase in adipose tissue and musculoskeletal discomfort characterize the normal aging process [1] and such changes lead to a decrease in physical activity, reduction in quality of life, loss of independence and development of co-morbidities such as worsening cardiovascular [2] and mental health [3]. National Health Interview Survey 2012 reported that three of the four most common medical conditions in the United States were musculoskeletal conditions, including lower back pain, chronic joint pain, and arthritis [4]. Studies reported higher rate of musculoskeletal health issues in rural areas than the US average [5] and a need for access to care in rural areas [6].

Sex differences in the biology of different organ system and the influence of sex hormones in modulating health and disease are increasingly relevant in clinical and research area, including musculoskeletal health. Gender may be a predictor of chronic musculoskeletal health with females at higher risk than males [4]. For example, de Graaf et al. recently reported there are significant difference in short musculoskeletal function assessment between men and women in the Dutch population [7]. In 2012, nearly 10.7 million women, compared with 7.5 million men, reported limitations in activities of daily living due to musculoskeletal conditions [4]. Other contextual variables (e.g., age, education, income), lifestyle/anthropometric variables (e.g., body mass index, height, physical activity), existing diseases (e.g., diabetes, stroke, chronic obstructive pulmonary diseases), and physical functioning impairment have been shown to contribute to walking speed, an indicator related to the musculoskeletal health, in the older US population [8]. In addition to population characteristics and socio-economic factors, blood chemistry parameters such as blood glucose, glycated hemoglobin (HbA1C), low-density lipoprotein (LDL), very-low-density lipoprotein (VLDL), C-reactive protein (CRP), and uric acid, are also highly associated with musculoskeletal health [9–13].

A recent cross-sectional study [13] reported that increased physical activity was associated with improvements in some metabolic and inflammatory markers of health [13]. On the other hand, increasing research effort has been focused on the role of insulin resistance in age-related conditions or geriatric syndromes, such as musculoskeletal morbidities. Based on a population-based National Health and Nutrition Examination Survey (NHANES), Kuo et al. [14] reported an inverse association between insulin resistance and habitual gait speed in non-diabetic older men (≥50 years), suggesting insulin resistance is an important indicator of gait function among men. Further, Kalyani et al. [15] reported that elevated fasting glucose level was associated with lower grip strength in older men, but not in women without self-reported diabetes and/or use of diabetes medication. Zhang et al. [8] also reported that poor lower extremity function was associated with pre-diabetes/diabetes in older Chinese. However, little is known the correlations between inflammatory and metabolic markers and musculoskeletal health-related parameters including hand grip strength, musculoskeletal discomfort, and gait disturbance in a rural setting, and the differences of such correlations due to gender difference. Such information would provide a base for future nutritional advice and life style education to manage musculoskeletal health in a rural population. Therefore, the current study investigated the factors affecting musculoskeletal health in terms of hand grip strength, musculoskeletal discomfort, and gait disturbance for different genders. We focused on a rural West Texas multiethnic adult and elderly cohort using data collected through the Project FRONTIER (Facing Rural Obstacles to healthcare Now Through Intervention, Education & Research) to explore the natural course of chronic disease development and its impact on longitudinal cognitive, physical, social, and interpersonal functioning in a multi-ethnic adult sample from rural communities of West Texas [16]. Study participants are followed over time to test for changes in physical, mental, and cognitive health and the factors that may influence those changes, in order to collect information for effective disease management and improvement of the overall health of individuals living in this geographic area and beyond. In summary, the objective of this study was to investigate the factors affecting musculoskeletal health in terms of hand grip strength, musculoskeletal discomfort, and gait disturbance for different genders in a rural West Texas multiethnic adult and elderly cohort.

## Methods

### Samples

Data were drawn from the participant database of the Project FRONTIER, an ongoing, community-based study of health and aging in rural West Texas, US, involving individuals 40 years and older, who were recruited and have gone through medical examination, neurophysiological and neuropsychological testing described by Johnson et al. [17]. The project covers the counties of Cochran, Bailey, and Parmer located on the Texas and New Mexico border [16]. Participants were recruited by community recruiters/assessors through multiple means including brochures/flyers, presentations, promotion events, in-person and/or door-to-door solicitation, and snowball recruitment. Since 2007, study participants have been seen every three years to go through a standardized medical examination with review of systems, detailed medical history review, Hachinski Ischemia Scale survey, neuropsychological assessment, and fasting blood test. Data are de-identified prior to storage. In the present study, only the first observation data were used in the analyses. Subjects who reported a history of diagnosed stroke, neurological disease, or movement disorder were excluded because these conditions could potentially affect coordination and gait. A total of 1117 participants (343 men and 774 women) were included in the analyses of the present study.

### Ethics and consent to participate

The current study was conducted under a protocol approved by the Institutional Review Board (IRB) of Texas Tech University Health Sciences Center (Study # L06-028), and all participants signed a written informed consent. All data used in the analysis had been de-identified.

### Medical history and measures

The questionnaires used in Project FRONTIER included the National Health Interview Survey (version 2008, http://www.cdc.gov/nchs/nhis.htm) and Behavioral Risk Factor Surveillance System [BRFSS] (version 2008, http://www.cdc.gov/brfss/about/about_brfss.htm). Demographic data, education, employment status, income, general health, medication intake, and existence of osteoarthritis were self-reported by participants. Based on questionnaire survey, participants who responded “Excellent”, “Very good” or “Good” general health were defined as in “Good or better health” condition, and those who responded “Fair” or “Poor” general health were categorized as in “Fair or poor health” condition [18]. Having a chronic health problem was defined as having at least one of the chronic health problems of diabetes, pulmonary disease, and heart disease, as reported by participants and confirmed with clinical laboratory and medical exam using consensus diagnosis by a physician and other health care professionals. Participants’ height and weight were measured by a nurse or an interviewer to calculate body mass index (BMI). Waist circumference was determined by abdominal girth, measured at the umbilicus while standing. Body fat was measured by bioelectrical impedance analysis using a hand-to-hand bioelectrical impedance meter (Omron Body Fat Analyzer HBF-306, Omron, Bannockbum, IL) [19].

Fasting blood sample was collected via venipuncture for lipid panel (LDL and VLDL), comprehensive metabolic panel, and measurement of blood glucose, HbA1c and CRP.

### Dominant hand grip strength, musculoskeletal discomfort, and gait disturbance

Hand grip strength was measured by a dynamometer (Smedley Hand Dynamometer 19117, Stoelting Co., Wood Dale, IL). Setting was adjusted for different hand size within the range between 3 (small hand) and 5 (large hand) based on individual participant’s preference. During the test, the participant was instructed to keep his/her arm extended with the dynamometer pointing toward the floor. The average of three trials for the dominant hand grip strength was recorded for the analysis, following the established procedure [20].

Musculoskeletal discomfort was self-reported by participants using a cumulative score with one point (answered “yes”) for each of the following categories: joint pain, back pain, swelling, stiffness, deformity, muscles aches and locked joints. Therefore, the lowest cumulative score was 0 and the highest cumulative score was seven. Participants were rated for the presence of gait disturbance from their medical examinations by the physician. Having “gait disturbance” was defined as the presence of at least one of the gait abnormalities including stiff, spastic, narrow-based, wide-based, unstable and shuffling gaits.

### Statistical analyses

Descriptive statistics were used to describe the characteristics of the study cohort. Mean and standard deviation (SD), or minimum, medium and maximum were calculated to characterize the distribution of variables of interest, as appropriate. Multivariable logistic regression was performed to explore the relationships between independent variables including demographics risk factors and biomarkers, and binary musculoskeletal health parameters related to gait disturbance. Multivariable ordinary linear regression and negative binomial regression were performed to assess how the risk factors and biomarkers were associated with grips strength and musculoskeletal discomfort, respectively. All the analyses were stratified by gender, and P values less than 0.025 were considered statistically significant. Analyses were performed using SAS software (Windows version 9.3; SAS Institute, Cary, NC).

## Results

### Participant characteristics

Descriptive statistics of participants are presented in Table 1. Of the 1143 participants, mean age for male (*n* = 343) and female (*n* = 774) were 60.5 years (SD 11.7) and 58.0 years (SD 12.3), respectively (*p* < 0.001). There were significant differences in uric acid (*p* < 0.001), waist circumference (*p* < 0.001), body fat (*p* < 0.001), and hand grip strength (*p* < 0.001) between males and females, showing males had higher uric acid level, larger waist circumference, and greater hand grip strength, while females had higher percentage body fat. There were differences in years of education (female > male, *p* = 0.043), income (male > female, *p* = 0.033), BMI (female > male, *p* < 0.001), and incidence of coronary artery disease (male > female, *p* = 0.001).

**Table 1:** Participant Characteristics

	Male (*n* = 343)		Female (*n* = 774)		*P* value
Parameter	Mean	SD	Mean	SD	
Age (years)	60.5	11.7	58.0	12.3	<0.001
C-reactive protein (mg/dL)	2.33	3.72	3.02	4.30	0.147
Glucose (mg/dL)	114.60	41.10	112.10	48.97	0.378
HbAc1 (%)	6.13	1.56	6.13	1.40	0.541
Uric acid (mg/dL)	6.21	1.41	4.97	1.35	<0.001
LDL (mg/dL)	111.15	37.09	113.70	35.08	0.283
vLDL (mg/dL)	36.25	27.26	32.03	18.43	0.062
Waist circumference (cm)	101.11	13.05	97.25	16.20	<0.001
Body fat (%)	28.61	6.78	39.07	7.01	<0.001
Hand grip strength (kg)	38.69	10.61	25.91	6.99	<0.001
	Medium	Min, max	Medium	Min, max	*P* value
Education (years)	10	0, 20	12	0, 20	0.043
Income per year ($10,000 increatment)	2–3	<1, >7	2–3	<1, >7	0.033
Musculoskeletal discomfort	1	0,7	1	0,7	0.607
	Frequency	Percentage	Frequency	Percentage	*P* value
BMI (kg/m^2^)					<0.001
Underweight (BMI < 18.5)	0	0 %	4	0.52 %	
Normal Weight (BMI 18.5–24.9)	57	16.72 %	136	17.59 %	
Overweight (BMI 25–29.9)	156	45.75 %	252	32.60 %	
Obese (BMI > 30)	128	37.54 %	381	49.29 %	
Race					0.466
Hispanic	190	55.39 %	455	58.86 %	
Non-Hispanic White	138	40.23 %	292	37.77 %	
Others	15	4.37 %	26	3.36 %	
Employment (yes)	98	47.57 %	192	41.03 %	0.114
Fair or poor Health^b^	134	39.07 %	268	34.63 %	0.154
Arthritis (yes)	26	7.58 %	67	8.66 %	0.548
Coronary Artery Disease (yes)	24	7.00 %	19	2.45 %	0.001
COPD	3	0.87 %	6	0.78 %	1.000
Chronic health problem^a^ (yes)	248	78.7 %	480	69.1 %	0.002
Diabetes (yes)	36	10.50 %	70	9.04 %	0.445
Hypertension (yes)	51	14.87 %	111	14.34 %	0.817
Metabolic Syndrome (yes)	2	0.58 %	3	0.39 %	0.646
Any cancer (yes)	10	2.92 %	28	3.62 %	0.550
Vitamin D deficiency (yes)	3	0.87 %	2	0.26 %	0.172
Fibromyalgia (yes)	2	0.58 %	8	1.03 %	0.732
Gait disturbance (yes)	18	6.19 %	59	8.94 %	0.151

^a^Participants with at least one of the chronic health problems of diabetes, pulmonary disease, and heart disease are defined as having chronic health problems

^b^Based on questionnaire survey, participants who responded “Excellent”, “Very good” or “Good” general health were defined as in “Good or better health” condition, and those who responded “Fair” or “Poor” general health were defined as in “Fair or poor health” condition

### Hand grip strength

Table 2 presents gender comparison of differences in dominant hand grip strength due to variations in different parameters. Data of estimated changes in dominant hand grip strength due to variations in different parameters, both categorical and continuous (quantitative), are exhibited. For instance, for the categorical variable of race, the dominant hand grip strength of non-Hispanic white males is estimated to be greater than that of Hispanic males by 4.5731 kg. For the continuous variable of age, the dominant hand grip strength decreases at a rate of 0.4475 kg per year in all males. In both genders, Hispanic had significant lower dominant hand grip strength than “Others”, with p values of 0.024 and 0.008 for males and females, respectively. However, this “Others” group accounts for only 4.37 % (*n* = 15) of all the participants, and may not truly represent all other races in the region. In both genders, hand grip strength decreased with age. Compared with participants with normal BMI, overweight females (BMI ≥ 25 kg/m^2^) tended to have greater hand grip strength (0.05 < *p* < 0.1), and obese males (BMI > 30 kg/m^2^) had marginally greater hand grip strength (*p* = 0.0503). Neither waist circumference nor body fat was an indicator of dominant hand grip strength (*p* > 0.05). Fair or poor health was significantly associated with lower hand grip strength in females (*p* < 0.001), while there was a similar trend in males (*p* = 0.0945). Participants with arthritis also had significantly lower hand grip strength regardless of gender. Higher LDL level was a predictor of greater hand grip strength in females (*P* = 0.013), but not in males.

**Table 2:** Gender comparison of differences in dominant hand grip strength due to variations in different parameters

Parameters	Male		Female	
	Estimated difference^a, b^	*p* value*	Estimated difference^a, b^	*p* value**
Race				
Non-Hispanic white vs. Hispanic	4.5731 kg^a^	0.0409	1.5061 kg^a^	0.1211
Others vs. Hispanic	8.0461 kg^a^	0.0243	4.4964 kg^a^	0.0075
Age (years)	–0.4475 kg/yr^b^	<0.0001	–0.2608 kg/yr^b^	<0.0001
BMI				
Underweight vs. Normal	----	---	–1.8212 kg^a^	0.5370
Overweight vs. Normal	2.9985 kg^a^	0.1555	1.4989 kg^a^	0.0947
Obese vs. Normal	5.7179 kg^a^	0.0503	2.1318 kg^a^	0.0671
Waist circumference (cm)	0.0030 kg/cm^b^	0.9706	0.0384 kg/cm^b^	0.1778
Body fat (%)	–0.1199 kg/(% body fat)^b^	0.4049	–0.1014 kg/%	0.1187
Income ($10,000 increment)	–0.0566 kg/$10K^b^	0.8795	0.2433 kg/$10K^b^	0.1375
Education (years)	–0.1034 kg/yr^b^	0.6059	0.0120 kg/yr^b^	0.9058
Employed				
Yes vs. No	–0.6770 kg^a^	0.6782	0.4578 kg^a^	0.5009
Chronic health problem^c^				
Yes vs. No	–1.5912 kg^a^	0.3011	–0.4255 kg^a^	0.5235
General health^d^				
Fair or poor health vs. Good or better health	–2.5250 kg^a^	0.0945	–3.8704 kg^a^	<.0001
Arthritis				
Yes vs. No	–6.5910 kg^a^	0.0089	–2.4360 kg^a^	0.0102
CRP (mg/dL)	0.1008 kg/(mg/dL)^b^	0.5909	0.0022 kg/(mg/dL)^b^	0.9784
Glucose (mg/dL)	–0.0212 kg/(mg/dL)^b^	0.2234	–0.0058 kg/(mg/dL)^b^	0.3646
HbA1c (%)	–0.3704 kg/%^b^	0.4586	–0.2888 kg/%^b^	0.2248
Uric acid (mg/dL)	–0.2043 kg/(mg/dL)^b^	0.7648	0.1553 kg/(mg/dL)^b^	0.6565
LDL (mg/dL)	0.0315 kg/(mg/dL)^b^	0.0859	0.0210 kg/(mg/dL)^b^	0.0128
vLDL (mg/dL)	0.0007 kg/(mg/dL)^b^	0.9817	0.0045 kg/(mg/dL)^b^	0.8264

*BMI* body mass index, *CRP* C-reactive protein, *HbA1c* glycated hemoglobin, *LDL* low density lipoprotein, *vLDL* very low density lipoprotein

^a^Difference in dominant hand grip strength due to the difference of a categorical variable

^b^Difference in dominant hand grip strength per unit change of a continuous variable

^c^Participants with at least one of the chronic health problems of diabetes, pulmonary disease, and heart disease are defined as having chronic health problems

^d^Based on questionnaire survey, patients who had “Excellent”, “Very good” or “Good” general health were defined as having “good or better health” condition, and those who had “Fair” or “Poor” general health were defined as having “fair or poor health” condition

*n = 177; from multivariable analysis; adjusted for demographic and socioeconomic factors; critical value is 0.025

**n = 379; from multivariable analysis; adjusted for demographic and socioeconomic factors; critical value is 0.025

### Musculoskeletal discomfort

Table 3 presents gender comparison of differences in musculoskeletal discomfort due to variations in different parameters. Data of estimated changes in musculoskeletal discomfort due to variations in different parameters are exhibited. For instance, for the categorical variable of race, non-Hispanic white female group is estimated to have more musculoskeletal discomfort than Hispanic female group by 42.5 % [exp (0.3542) – 1]. For the continuous variable of age, musculoskeletal discomfort increases at a rate of 1.2 % [exp (0.0122) – 1] per year in all females. Advanced age was a marginally significant predictor of musculoskeletal discomfort in females (*p* = 0.0254), but not in males (*p* = 0.5748). Relative to females with normal weight, underweight females tended to report less musculoskeletal discomfort (*p* = 0.0596). Greater waist circumference was associated with greater musculoskeletal discomfort in males only (*p* = 0.005), not in females (*p* = 0.1360). Participants with at least one of the chronic health problems of diabetes, pulmonary disease, and heart disease were associated with greater musculoskeletal discomfort in females (*p* = 0.0038) and a trend in males (*p* = 0.0836). In both genders, fair or poor health and report of “arthritis” were predictors of greater musculoskeletal discomfort.

**Table 3:** Gender comparison of differences in musculoskeletal discomfort due to variations in different parameters

	Male		Female	
Parameters	Estimated difference^a, b^	*p* value*	Estimated difference^a, b^	*p* value**
Race				
Non-Hispanic white vs. Hispanic	–0.0396^a^	0.8634	0.3542^a^	0.0286
Others vs. Hispanic	–0.5542^a^	0.1379	0.5496^a^	0.0188
Age (years)	0.0041/yr^b^	0.5748	0.0122/yr^b^	0.0254
BMI				
Underweight vs. Normal	---	---	–1.4221	0.0596
Overweight vs. Normal	0.0165	0.9400	0.1642	0.2844
Obese vs. Normal	–0.2487	0.4159	0.1838	0.3354
Waist circumference (cm)	0.0248/cm^b^	0.0052	0.007/cm^b^	0.1360
Body fat (%)	–0.0100/(% body fat)^b^	0.4675	0.0009/%^b^	0.9359
Income ($10 K increment)	–0.0143/$10K^b^	0.7162	–0.0367/$10K^b^	0.1876
Education (years)	–0.0237/yr^b^	0.2348	0.0197/yr^b^	0.2287
Employed				
Yes vs. No	0.0354	0.8289	0.1823	0.1006
Chronic health problems^c^				
Yes vs. No	0.276	0.0836	0.3377	0.0038
General health^d^				
Fair or poor health vs. Good or better health	0.4054	0.0069	0.5678	<.0001
Arthritis				
Yes vs. No	0.6651	0.0011	0.5267	<.0001
CRP (mg/dL)	–0.0238/(mg/dL)^b^	0.2825	0.0004/(mg/dL)^b^	0.9721
Glucose (mg/dL)	0.0005/(mg/dL)^b^	0.7734	–0.0009/(mg/dL)^b^	0.3888
HbA1c (%)	0.0084/%^b^	0.8662	–0.0411/%^b^	0.2931
Uric acid (mg/dL)	–0.0421/(mg/dL)^b^	0.4885	–0.0010/(mg/dL)^b^	0.9817
LDL (mg/dL)	–0.0015/(mg/dL)^b^	0.4046	–0.0023/(mg/dL)^b^	0.0884
vLDL (mg/dL)	–0.0026/(mg/dL)^b^	0.3602	0.0003/(mg/dL)^b^	0.9161

*BMI* body mass index, *CRP* C-reactive protein, *HbA1c* glycated hemoglobin, *LDL* low density lipoprotein, *vLDL* very low density lipoprotein

^a^Logarithmic (*ln*) value of difference in musculoskeletal discomfort due to the change in a categorical variable

^b^Logarithmic (*ln*) value of difference in musculoskeletal discomfort per unit change of a continuous variable

^c^Participants with at least one of the chronic health problems of diabetes, pulmonary disease, and heart disease are defined as having chronic health problems

^d^Based on questionnaire survey, patients who had “Excellent”, “Very good” or “Good” general health were defined as having “good or better health” condition, and those who had “Fair” or “Poor” general health were defined as having “fair or poor health” condition

*n = 167; from multivariable analysis; adjusted for demographic and socioeconomic factors; critical value is 0.025

**n = 367; from multivariable analysis; adjusted for demographic and socioeconomic factors; critical value is 0.025

### Gait disturbance

Table 4 compares the numbers and percentages of male and female participants with gait disturbance, as well as the corresponding odds ratio values for different parameters. In the present cohort, 6 % males and 9 % females were observed having gait disturbance. Although some raw odds ratio values show possible associations of parameters with gait disturbance, such associations disappear after the odd ratio is adjusted for demographic and socioeconomic factors including race, chronic health problem, arthritis, employment, income, and waist circumference.

**Table 4:** Comparison of occurrence of gait disturbance by gender^a^

	Male				Female			
	Number of participants (%)		Odds Ratio (CI)		Number of participants (%)		Odds Ratio (CI)	
Parameters	Normal	Gait disturbance	Odds Ratio	Odds Ratio^b^	Normal	Gait disturbance	Odds Ratio	Odds Ratio^c^
	(*n* = 273)	(*n* = 18)	(raw)	(adjusted)	(*n* = 601)	(*n* = 59)	(raw)	(adjusted)
Race								
Hispanic	153 (56.04)	11 (61.11)			374 (62.33)	21 (35.59)		
Non-Hispanic white	108 (39.56)	5 (27.78)	0.64 (0.22,1.91)	0.29 (0.03,3.42)	207 (34.50)	34 (57.63)	2.93 (1.65,5.17)	1.17 (0.24,5.72)
Others	12 (4.40)	2 (11.11)	2.32 (0.46,11.68)	0.42 (0.03,5.36)	19 (3.17)	4 (6.78)	3.75 (1.17,12.01)	0.67 (0.07,6.85)
BMI								
Underweight	---	---	not estimable	not estimable	4 (0.67)	0 (0.00)	not estimable	not estimable
Normal	46 (16.91)	5 (27.78)			109 (18.14)	12 (20.69)		
Overweight	123 (45.22)	7 (38.89)	0.52 (0.16,1.73)	0.52 (0.08,3.37)	198 (32.95)	12 (20.69)	0.55 (0.24,1.27)	0.51 (0.14,1.85)
Obese	103 (37.87)	6 (33.33)	0.54 (0.16,1.85)	0.31 (0.02,5.73)	290 (48.25)	34 (58.62)	1.06 (0.53,2.13)	0.43 (0.08,2.19)
Chronic health problem^d^								
No	61 (22.34)	4 (22.22)			207 (34.50)	7 (11.86)		
Yes	212 (77.66)	14 (77.78)	1.01 (0.32,3.17)	1.48 (0.32,6.98)	393 (65.50)	52 (88.14)	3.91 (1.75,8.77)	2.08 (0.66,6.51)
General health^e^								
Good or better health	177 (64.84)	5 (27.78)			385 (64.06)	31 (52.54)		
Fair or poor health	96 (35.16)	13 (72.22)	4.79 (1.66,13.85)	4.65 (1.04,20.82)	216 (35.94)	28 (47.46)	1.61 (0.94,2.76)	3.67 (1.17,11.47)
Arthritis								
No	253 (92.67)	13 (72.22)			557 (92.68)	40 (67.80)		
Yes	20 (7.33)	5 (27.78)	4.87 (1.58,15.02)	3.21 (0.60,17.21)	44 (7.32)	19 (32.20)	6.01 (3.21,11.25)	2.58 (0.96, 6.94)
Employed								
No	78 (49.37)	13 (92.86)			211 (57.65)	36 (85.71)		
Yes	80 (50.63)	1 (7.14)	0.08 (0.01,0.59)	0.47 (0.08,2.79)	155 (42.35)	6 (14.29)	0.23 (0.09,0.55)	0.55 (0.17,1.82)
	Median (min, max)	Median (min, max)			Median (min, max)	Median (min, max)		
Age (yrs)	60.0 (40.0,93.0)	72.5 (47.0,87.0)	1.08 (1.03,1.13)	1.07 (0.99,1.15)	55.0 (40.0,94.0)	72.0 (41.0,96.0)	1.08 (1.06,1.11)	1.06 (1.01,1.12)
Education (years)	10.0 (0.0,20.0)	8.5 (0.0,16.0)	0.94 (0.85,1.03)	1.07 (0.88,1.30)	11.0 (0.0,20.0)	12.0 (1.0,20.0)	1.03 (0.96,1.09)	1.13 (0.95,1.33)
Income ($10,000 increment)	3.0 (1.0,8.0)	2.0 (1.0,8.0)	0.77 (0.58,1.02)	0.85 (0.53,1.36)	3.0 (1.0,8.0)	2.0 (1.0,8.0)	0.77 (0.66,0.91)	0.86 (0.66,1.12)
Waist circumference (cm)	99.1 (78.7,149.9)	99.1 (86.4,142.2)	1.02 (0.93,1.12)	1.19 (0.93,1.52)	96.5 (50.8,210.8)	104.1 (66.0,251.5)	1.07 (1.03,1.11)	1.02 (0.93,1.12)
Body fat (%)	29.3 (0.0,43.3)	29.9 (0.0,44.4)	0.99 (0.92,1.06)	0.91 (0.81,1.01)	40.0 (0.0,53.2)	42.9 (0.0,48.4)	1.03 (0.98,1.09)	1.07 (0.96,1.19)
CRP (mg/dL)	1.0 (0.2,33.4)	1.6 (0.5,12.4)	1.01 (0.89,1.14)	1.07 (0.93,1.24)	1.1 (0.1,31.1)	4.3 (0.5,24.2)	1.12 (1.06,1.18)	1.09 (1.01,1.19)
Glucose (mg/dL)	102.0 (65.0,304.0)	103.0 (83.0,303.0)	1.01 (1.00,1.02)	1.01 (1.00,1.03)	97.0 (67.0,520.0)	98.0 (75.0,400.0)	1.00 (1.00,1.01)	1.01 (1.00,1.01)
HbA1c (%)	5.7 (4.6,14.3)	6.1 (4.8,13.0)	1.26 (0.99,1.61)	1.49 (0.99,2.24)	5.7 (4.1,14.0)	6.0 (4.7,14.0)	1.13 (0.97,1.31)	1.32 (1.01,1.70)
Uric acid (mg/dL)	6.0 (2.2,10.5)	6.1 (4.1,8.3)	0.94 (0.65,1.37)	0.98 (0.55,1.75)	4.7 (1.8,10.8)	5.2 (2.5,9.7)	1.29 (1.02,1.62)	1.00 (0.69,1.44)
LDL (mg/dL)	109.0 (34.0,253.0)	90.5 (49.0,201.0)	1.00 (0.98,1.01)	1.00 (0.98,1.02)	112.0 (38.0,250.0)	105.0 (24.0,190.0)	0.99 (0.98,0.99)	0.99 (0.98,1.01)
vLDL (mg/dL)	29.0 (9.0,235.0)	30.0 (16.0,58.0)	0.99 (0.97,1.02)	1.00 (0.98,1.03)	28.0 (9.0,139.0)	25.0 (9.0,230.0)	1.01 (0.99,1.02)	1.01 (0.99,1.03)

*BMI* body mass index, *CRP* C-reactive protein, *HbA1c* glycated hemoglobin, *LDL* low density lipoprotein, *vLDL* very low density lipoprotein

^a^Patients with at least one of the gait abnormalities including stiff, spastic, narrow-based, wide-based, unstable and shuffling gaits are defined as having gait disturbance

^b^ *n* = 159 due to missing values; from multivariable analysis; adjusted for demographic and socioeconomically factors

^c^n = 362 due to missing values; from multivariable analysis; adjusted for demographic and socioeconomically factors

^d^Patients with at least one of the chronic health problems of diabetes, pulmonary disease, and heart disease are defined as having chronic health problems

^e^Based on questionnaire survey, patients who had “Excellent”, “Very good” or “Good” general health were defined as having “good or better health” condition, and those who had “Fair” or “Poor” general health were defined as having “fair or poor health” condition

Both males and females with fair or poor health had significantly higher risk of gait disturbance than those good or better health; and the adjusted OR values were 4.65 (95 % CI: 1.04, 20.82) and 3.67 (95 % CI: 1.17, 11.47) for males and females, respectively. In females, the risk of gait disturbance increases by 6 % per year of age, while this relationship was not significant in males. In females, both higher CRP and HbA1C levels were significantly associated with higher risk of gait disturbance, while this relationship was not significant in males.

## Discussion

This is the first cohort study to evaluate associations between population characteristics, socio-economic factors, blood chemistry parameters, and musculoskeletal health (hand grip strength, musculoskeletal discomfort, and gait disturbance) by gender. Our rural West Texas population exhibits unique demographic characteristics compared to general population in terms of higher percentage of Hispanic population, higher obesity rate, higher poverty rate, higher unemployment rate, and higher prevalence of chronic diseases, in agreement with results of published studies [21, 22]. In our cohort, 55.4 % of the males and 58.9 % of the females were Hispanic. Compared to non-Hispanic white and others females, Hispanic females had greater musculoskeletal discomfort after adjusting for demographic and socioeconomic factors. Such findings are consistent with Quiben’s study [7] that older Mexican Americans have poorer functional health (as assessed by walking speed) than European Americans.

Socioeconomic status and education were not associated with dominant hand grip strength, musculoskeletal discomfort, or gait disturbance in our rural multiethnic cohort, regardless of gender. This finding is different from the United States national data that health related quality of life in the elderly, as assessed by overall functional health, is positively associated with household income [23]. This discrepancy may be due to the difference in age that our participants’ mean age was about 60 years old compared to 73 years old for the study of Huguet et al. [23]. Further research addressing the elderly population in other rural areas would be valuable to assess if such disparity exist as a general rule.

The present results show that age was an important non-modifiable risk factor for musculoskeletal health decline (i.e., hand grip strength, musculoskeletal discomfort (females only), and gait disturbance (females only), particularly in females. Hand grip strength is known to be associated with functioning in other muscle groups and activities of daily living, as well as incident disability [24]. By stratifying our study cohort by gender, we found that age was an important negative predictor of hand grip strength in both genders and such findings were consistent with previous studies that hand grip strength was strongly and inversely associated with age.

Age was also a marginally significant risk factor for musculoskeletal discomfort and a significant risk factor for gait disturbance in females. The present result agrees with DePalma et al. [25] in that age was associated with significant increase in chronic low back pain in females. Our findings of a positive correlation between age and gait disturbance is also in agreement with Wilson et al. [26] that gait disorder and rigidity are usually progressive with age. Krasovsky et al. [27] investigated gait stability and inter-limb coordination following a perturbation to the dominant leg during walking at comfortable speed, and found that older male adults had a longer period of initial destabilization, took a longer time to recover center of mass stability and double-support duration, and had larger whole body long-lasting phase shifts, suggesting greater risk of gait disturbance than younger male adults. As expected, the aging population is prone to higher rates of nearly all musculoskeletal conditions than younger population. In large part, these conditions can be attributed to wear and tear of bones and joints over a lifetime [4].

Our study suggests that waist circumference was positively correlated with musculoskeletal discomfort in males only. The unit for waist circumference measurement was cm, and for every unit increase in waist circumference, there was a 2.5 % increase ([exp (0.0248)-1], Table 3) in musculoskeletal discomfort rating. Waist circumference is a measure of abdominal obesity. The association between obesity and osteoarthritis is well-established, especially for weight-bearing joints [28], because these joints are used more intensely in rural areas, where people have less access to health care, resulting in greater pain and disability [29]. Moreover, a study has shown that a waist circumference is highly associated with dynapenia, age-associated impairment of muscle strength [30]. A higher percentage of physical inactivity was observed in our rural cohort (27 %) than the Texas average (23 %) [31]. On the other hand, the current study revealed that presence of chronic health problems (having at least one of the chronic health problems of diabetes, pulmonary disease, and heart disease) in females was a significant predictor for musculoskeletal discomfort. Women with chronic health problems reported 40 % higher ([exp (0.3377)-1], Table 3) musculoskeletal discomfort ratings. A recent study by Pelaez-Ballesta [32] concluded that older age, being female, disability, and having had physically demanding work were associated with a greater likelihood of having a musculoskeletal pain. Miro et al. [33] also commented that the prevalence of any pain was similar across age groups but higher in females, and among individuals suffering from pain, 94.2 % reported chronic pain (i.e., pain of more than three months’ duration). Furthermore, several studies have suggested that women are also more sensitive to pain [34, 35]. This may explain why women reported greater musculoskeletal discomfort in our study cohort.

The status of general health was an important predictor of musculoskeletal health in our cohort. For example, the present study showed that participants with fair or poor health were associated with inferior musculoskeletal health, i.e., weaker dominant hand grip strength (females only), greater musculoskeletal discomfort, and higher risk of gait disturbance. The findings of the correlation of overall general health and musculoskeletal health are supported by a previous study showing gait disturbance associated with cardiovascular decline and greater mortality [36].

Interestingly, we found that higher CRP concentration was positively associated with risk for gait disturbance in females (1.09 adjusted odds ratio), a result supported by Arts et al. [37] who reported that performance-based physical frailty (encompassing gait speed, hand grip strength, and low physical activity) was associated with higher levels of inflammatory markers (i.e., CRP, interleukin-6). Moreover, a positive association was found between HbA1c level and gait disturbance in females only; i.e., females have a 32 % increase in risk of gait disturbance per 1 % increase in HbA1c level. This result is supported by Arvanitakis et al. [38] who found that diabetes (defined as HbA1c ≥ 7 %) was associated with worsening gait disturbance in the elderly.

There are several limitations to this study. First, musculoskeletal discomfort was self-reported by participants using a cumulative score with one point for each of the following categories: joint pain, back pain, swelling, stiffness, deformity, muscles aches and locked joints. Information regarding individual pain category was dichotomous (yes/no) collected during the medical examination review of systems without any detailed information regarding the degree of pain. This combination was based on a review of the interview items tapping elements of musculoskeletal-related pain and a decision to weight them equally when summing the number of different complaints endorsed by each participant. Authors made it as a logical decision to come up with a summary measure. The same reasoning was used for creating “gait disturbance” defined as the presence of at least one of the gait abnormalities including stiff, spastic, narrow-based, wide-based, unstable and shuffling gaits. The gait determinations were made by physicians as part of the standardized medical exam and possibly being drawn from interview items. Both assessments placed less emphasis on analyzing specific musculoskeletal conditions versus generally abnormal musculoskeletal health. There is no independent validation of this combined measure of musculoskeletal discomfort and gait disturbance. Future studies are warranted to test for replicable results across populations which would then support its validity.

Second, the cross-sectional nature of the present study prohibits any causal conclusion. Third, other factors (e.g., dietary intake and physical activity patterns) may exist between genders accounting for hand grip strength, musculoskeletal discomfort, and gait functioning analyzed in this study. Despite these weaknesses, the current preliminary study paves the way for a more in-depth study of community musculoskeletal health by gender. While the present work was a cross-sectional study focusing on hand grip strength, musculoskeletal discomfort, and gait disturbance, future research should include longitudinal studies with interventions to improve musculoskeletal health targeting weight control and chronic disease prevention by gender in rural populations.

Although population aging is driving the epidemic of chronic diseases worldwide, substantial potential exists to modify the overall health decline associated with aging thereby reducing the burden of disease that arises mainly from disability and long-term care health expenditure [39]. This is of importance as more than one third of our participants reported to be in fair or poor health, and that rural-urban disparities in quality of life persist and less access to care in rural areas may contribute to these disparities [40]. Research outcomes support the value of community-based health promotion programs in rural areas, incorporating multidisciplinary health team and culturally competent materials to help elderly rural inhabitants enjoy better health and quality of life [41]. Such programs could be valuable in rural areas of West Texas, and the results of the present study may contribute to the design of the musculoskeletal health aspect of the program.

## Conclusion

Age, report of “arthritis”, fair or poor health and selected biomarkers indicative of systemic inflammation were negatively associated with musculoskeletal health in a rural multiethnic cohort. Males with greater waist circumference had greater musculoskeletal discomfort. In females, fair or poor health, advanced age, chronic health problems, C-reactive protein, and HbA1c was negatively associated with musculoskeletal health, where the association was not seen in males. Improving population overall health and educating on healthy lifestyle in regard to exercise and diet could be investigated for their effect on musculoskeletal health. Further rural and multiethnic prospective studies are warranted.

## Availability of data and materials

Project FRONTIER data is available to researchers by contacting the F. Marie Hall Institute for Rural and Community Health [Attn. Cathy Hudson], Texas Tech University Health Sciences Center 3601 4th Street, STOP 6232, Lubbock, Texas 79430–6232 and completing a Data Use Request and Agreement. Project FRONTIER data will then be provided in digital format at no cost except for shipping and handling.

## Abbreviations

BMI: body mass index
CRP: C-reactive protein
HbA1C: glycated hemoglobin
LDL: low-density lipoprotein
VLDL: very-low-density lipoprotein
